# Multi-view singular value decomposition for disease subtyping and genetic associations

**DOI:** 10.1186/1471-2156-15-73

**Published:** 2014-06-17

**Authors:** Jiangwen Sun, Jinbo Bi, Henry R Kranzler

**Affiliations:** 1 Department of Computer Science and Engineering, University of Connecticut, 371 Fairfield Way, Storrs, CT 06269, USA; 2 Treatment Research Center, University of Pennsylvania Perelman School of Medicine, Philadelphia, PA 24105, USA

**Keywords:** Genotype-phenotype association, Multi-view data analysis, Subtyping, Biclustering, Matrix decomposition

## Abstract

**Background:**

Accurate classification of patients with a complex disease into subtypes has important implications for medicine and healthcare. Using more homogeneous disease subtypes in genetic association analysis will facilitate the detection of new genetic variants that are not detectible using the non-differentiated disease phenotype. Subtype differentiation can also improve diagnostic classification, which can in turn inform clinical decision making and treatment matching. Currently, the most sophisticated methods for disease subtyping perform cluster analysis using patients’ clinical features. Without guidance from genetic information, the resultant subtypes are likely to be suboptimal and efforts at genetic association may fail.

**Results:**

We propose a multi-view matrix decomposition approach that integrates clinical features with genetic markers to detect confirmatory evidence for a disease subtype. This approach groups patients into clusters that are consistent between the clinical and genetic dimensions of data; it simultaneously identifies the clinical features that define the subtype and the genotypes associated with the subtype. A simulation study validated the proposed approach, showing that it identified hypothesized subtypes and associated features. In comparison to the latest biclustering and multi-view data analytics using real-life disease data, the proposed approach identified *clinical subtypes* of a disease that differed from each other more significantly in the genetic markers, thus demonstrating the superior performance of the proposed approach.

**Conclusions:**

The proposed algorithm is an effective and superior alternative to the disease subtyping methods employed to date. Integration of phenotypic features with genetic markers in the subtyping analysis is a promising approach to identify concurrently disease subtypes and their genetic associations.

## Background

For complex diseases, such as substance dependence or psychiatric disorders, a variety of clinical features that collectively indicate or characterize the disease phenotype often vary substantially among individuals [1]. Studies of genetic association or those that aim to match patients with certain treatments for a complex disease can be impeded by this phenotypic heterogeneity [2]. Case-control association studies based on a binary trait, such as the diagnosis of a disease, which partitions the population into cases (subjects with the disease) and non-cases (subjects without the disease), cannot differentiate the heterogeneous manifestations of the disease. Although many candidate genes or genomic regions have been associated with complex diseases [3], the characteristics or subtypes of the disease for which the association exists remain to be specified. For instance, the specific addictive behaviors that underlie the associations with candidate genetic variants need to be elucidated to clarify the risk for addiction [4].

Classification of a complex disease into homogeneous subcategories or subtypes may help to identify the genetic variants contributing to the effect of the subphenotypes [5,6]. However, prior studies have been limited to unsupervised cluster analysis or latent class analysis on clinical features to derive subtypes. Genotypic data have only been used to evaluate the validity of subtypes, such as in subsequent association tests with the derived subtypes, rather than to guide the creation of the subtypes. Consequently, the resultant subtypes may be of limited utility in genetic association analysis. Integration of data from both clinical and genomic dimensions also offers opportunities to find confirmatory evidence of a subtype based on both its genetic and clinical features. A few studies have examined the joint use of gene expression and genotypic data for cancer subtyping [7,8], but they did not identify a variable subspace (or a subset of features) in each data source so as to group subjects consistently across the two subspaces. Hence, they could not detect genetic variants associated with the identified clusters.

There has also been little research on this topic in the statistics literature. The most relevant area involves co-clustering [9] or multi-view data analysis [10], where samples are characterized or viewed in multiple ways, thus creating multiple sets of input variables. There are two types of co-clustering methods: (1) biclustering, also called two-mode clustering [11,12], which simultaneously clusters the rows and columns of a data matrix and (2) multi-view co-clustering [9,13], which seeks groupings that are consistent across different views. Biclustering is similar to another set of algorithms that search for subspaces and group subjects differently in each subspace [14].

Biclustering and subspace searching essentially identify different subgroups of subjects using different features (or markers), thus helping to identify genetic variants specific to a particular subgroup. However, this method can only be applied to one data matrix from a single view rather than data jointly from multiple views. Multi-view co-clustering, on the other hand, seeks a grouping of subjects that is consistent across different views (i.e., different sets of features), but the resultant clusters are defined using all of the available features, e.g., all of the studied genetic markers. Hence, it cannot be used to identify subtype-specific variants/features. Thus, to address our subtyping problem, we not only partitioned subjects in such a way that the subgroups differed in both clinical features and genetic markers, but also included a subspace search to identify the specific features or markers that defined the subgroups.

In this paper, we propose a multi-view matrix decomposition approach based on the sparse singular value decomposition (SSVD) technique [12] to classify a complex disease into subtypes using data both from the clinical and genetic views. The objective of this problem is to identify subject clusters that agree in the clinical and genetic views, and simultaneously identify features and markers that are associated with the clusters. Employing the *sparse* SVD in our approach is critical to its success, especially in terms of successfully detecting associated variants given that the number of truely associated variants are much fewer than the number of single nucleotide polymorphisms (SNPs) in the whole genome. The proposed approach was validated on synthetic datasets that were simulated to have subtype structures and several genetic markers associated with the subtypes and a real world clinical dataset that was aggregated from multiple genetic studies of substance dependence. We compared our approach to a biclustering approach [12] and the latest multi-view data analytics methods [9]. The results clearly show that the performance of our approach is superior to that of all other available methods.

## Methods

We start with a presentation of the notations that are used throughout the paper. A vector is denoted by a bold lower case letter as in **v** and ∥**v**∥_*p*_ represents its *ℓ*_*p*_-norm, which is defined by ∥**v**∥_*p*_=(|**v**_(1)_|^*p*^+⋯+|**v**_(*d*)_|^*p*^)^1/*p*^, where **v**_(*j*)_ is the *j*-th component of **v** and *d* is the length of **v**, i.e., the total number of components in **v**. We use ∥**v**∥_0_ to represent the so-called *0-norm* of **v** that equals the number of non-zero components in **v**. Denote **u**⊙**v** the component-wise (Hadamard) products of **u** and **v**. The set ${ℬ}_{d}$ contains all binary vectors of length *d*. A binary vector is a vector whose components equal either 0 or 1. A matrix is denoted by a bold upper case letter, e.g., **M**_*n*×*d*_ is a *n*-by-*d* matrix, and ∥**M**∥_*F*_ is its Frobenius norm defined by (*tr*(**M**^*T*^**M**))^1/2^ where *tr*(·) is the trace of a matrix. Rows and columns in **M** are denoted by **M**_(*i*,·)_ and **M**_(·,*j*)_, respectively.

### Review of single-view biclustering

We briefly review the biclustering method with a single view of data based on the sparse singular value decomposition [12]. For a single data matrix **M** of size *n*-by-*d*, a subgroup of its rows and a subgroup of its columns can be simultaneously obtained by the SSVD. The SSVD requires both the left and right singular vectors to be sparse. Let **u** of size *n* and **v** of size *d* be a pair of singular vectors resulting from the SSVD. Their outer product forms a sparse low-rank approximation of the original matrix, i.e., **M**=*σ***uv**^*T*^ where *σ* is the corresponding singular value. Then, the rows in **M** that correspond to non-zero components in **u** form a row subgroup. The columns in **M** that correspond to non-zero components in **v** form a column subgroup. The resultant row and column clusters help to define one another. The SSVD finds all singular vectors sequentially by repeatedly solving the following problem with a data matrix **M**:

(1) $$\begin{array}{ll} \;\underset{\sigma ,u,v}{\min} & \;∥M-\sigma uv^{T}{∥}_{F}^{2}+\lambda_{u}∥\sigma u{∥}_{0}+\lambda_{v}∥\sigma v{∥}_{0}\\ \text{subject to} & ∥u{∥}_{2}=1,∥v{∥}_{2}=1. \end{array}$$

The regularization terms ∥*σ***u**∥_0_ and ∥*σ***v**∥_0_ are used to enforce the sparsity of **u** and **v**. Note that the scalar *σ* will not affect the value of the regularization terms. The parameters *λ*_*u*_ and *λ*_*v*_ are two hyper-parameters to balance the approximation performance and the regularization terms. If both *λ*_*u*_ and *λ*_*v*_ equal 0, the optimal solution to this problem is the left and right singular vectors of **M** that correspond to its largest singular value. An alternating algorithm has been proposed in [12] to solve this problem effectively when *λ*_*u*_ and *λ*_*v*_ are not 0. This algorithm first initiates **u** and **v** by the first left and right singular vectors of **M**, then alternates between solving two sub-problems until it converges. The two sub-problems are: (a), fix **u** and find **v** that optimizes the objective of Eq.(1); (b), fix **v** and find **u** that optimizes the objective of Eq.(1).

Assume that each row of **M** represents a subject and each column corresponds to a feature. Once a pair of vectors **u** and **v** is obtained, a subject (row) cluster as indicated by the non-zero components of **u** is obtained. At the same time, the features on which the subjects in the cluster show high similarity are also identified in a column cluster as indicated by the non-zero components of **v**. More clusters can be obtained by repeating the optimization process with modified data matrices. To obtain subsequent clusters that are disjoint from any identified cluster in terms of subjects, the SSVD solves Eq.(1) using a new matrix **M** that excludes subjects (rows) already included in a row cluster. To obtain subsequent clusters that allow overlapping of subjects with identified clusters, the SSVD can solve Eq.(1) with the deflated **M**=**M**−*σ***uv**^*T*^ that removes the identified SVD components as used in the standard SVD.

### The proposed formula for two-view joint biclustering

In this section, we extend the single-view SSVD to find a consistent grouping of subjects across two data matrices. In a later section, the resulting method will be extended to incorporate more than two data matrices.

Assume that two data matrices denoted by **M**_1_ of size *n*-by- *d*_1_ and **M**_2_ of size *n*-by- *d*_2_ characterize the same set of *n* subjects from two different views. We can obtain **u**_1_, **v**_1_, and **u**_2_, **v**_2_ by a separate SSVD of **M**_1_ and **M**_2_, respectively. However, it will not guarantee that the row clusters specified by **u**_1_ and **u**_2_ agree. To make them consistent, **u**_1_ and **u**_2_ must have non-zero components at the same position. Note that the two **u** vectors are not necessarily the same, because they may be derived from very different features in the views, such as real-valued clinical features but discrete values in genetic markers.

We propose to use a binary vector **z** of size *n* that serves as a common factor to link the two views. Each component of **u** is then multiplied by the corresponding component of **z**, i.e., *u*_*i*_=*u*_*i*_*z*_*i*_. In other words, we represent each **u** vector by **z**⊙**u** in the objective function of SSVD to construct the sparse, rank one approximation matrices of **M**_1_ and **M**_2_, simultaneously. When **z** is sparse, both **z**⊙**u**_1_ and **z**⊙**u**_2_ will be sparse. Thus, we enforce the sparsity of **z** rather than individual **u** and solve the following optimization problem:

(2) $$\begin{array}{l} \underset{z,\sigma_{i},u_{i},v_{i},i=1,2}{\min}\;∥M_{1}\;-\sigma_{1}(z⊙u_{1})v_{1}^{T}{∥}_{F}^{2}\;+∥M_{2}\;-\sigma_{2}(z⊙u_{2})v_{2}^{T}{∥}_{F}^{2}\\ \;+\lambda_{z}∥z{∥}_{0}+\lambda_{v_{1}}∥\sigma_{1}v_{1}{∥}_{0}+\lambda_{v_{2}}∥\sigma_{2}v_{2}{∥}_{0},\\ \;\text{subject to}\;∥u_{i}{∥}_{2}=1,∥v_{i}{∥}_{2}=1,i=1,2,\\ \;z\in {ℬ}_{n}. \end{array}$$

where *λ*_*z*_, $\lambda_{v_{1}}$ and $\lambda_{v_{2}}$ are tuning parameters that balance the approximation errors and regularization terms. Although the values of **u**’s are constrained to be unit vectors, the values of **z**⊙**u**’s are not necessarily unit vectors. However, a careful examination reveals that for any optimal solution $\hat{u}$, we can find another optimal solution $\bar{u}$ that has non-zero values only at the entries indicated by the binary vector **z**, which ensures that $z⊙\bar{u}$ is also a unit vector. We first set ${\bar{u}}_{\left(j\right)}={\hat{u}}_{\left(j\right)}$, if **z**_(*j*)_≠0, or ${\bar{u}}_{\left(j\right)}=0$ otherwise, for *j*=1⋯,*n*. We then update the corresponding singular value $\sigma =\sigma ∥\bar{u}{∥}_{2}$ and rescale $\bar{u}=\bar{u}/∥\bar{u}{∥}_{2}$. This new vector $\bar{u}$ satisfies the constraints of Eq.(2), and together with the new *σ* will produce the same objective value as the original solution $\hat{u}$, thus corresponding to an optimal solution as well. We design a fast algorithm in a later section to find such a sparse $\bar{u}$ for Eq.(2).

We discuss two alternatives to the proposed formula (2). A restricted version of Eq.(2) may require **u**_1_=**u**_2_=**u** and then replace **z**⊙**u**_1_ and **z**⊙**u**_2_ by the same **u** in the objective function of Eq.(2), which leads to the following problem

(3) $$\begin{array}{l} \underset{\sigma_{i},u,v_{i},i=1,2}{\min}\;∥M_{1}-\sigma_{1}uv_{1}^{T}{∥}_{F}^{2}+∥M_{2}-\sigma_{2}uv_{2}^{T}{∥}_{F}^{2}\\ \;+\lambda_{u}∥u{∥}_{0}+\lambda_{v_{1}}∥\sigma_{1}v_{1}{∥}_{0}+\lambda_{v_{2}}∥\sigma_{2}v_{2}{∥}_{0},\\ \;\text{subject to}\;∥u{∥}_{2}=1,∥v_{i}{∥}_{2}=1,i=1,2. \end{array}$$

By requiring **u** to be sparse, it can also identify consistent row clusters between two views. The resultant optimization problem is easier to solve without integer variables in **z**. However, it is an unnecessarily stringent constraint to limit the search space to **u**_1_=**u**_2_, which rules out a number of potential solutions that may include the optimal row clusters. Another alternative is to minimize the difference between **u**_1_ and **u**_2_, which suffers from the same over-constrained problem because the exact values of the difference are not involved. Our problem only seeks to identify the indicators of whether or not a component of **u** is zero.

It is also useful to discuss the relation between Eq.(3) and the feature concatenation method, which simply merges the features from the two views in a cluster analysis. The feature concatenation method finds a single set of **u** and **v** for the data matrix [**M**_1_**M**_2_] by solving the following problem

(4) $$\begin{array}{l} \underset{\sigma ,u,v}{\min}\;∥[M_{1}\;M_{2}]-\sigma uv^{T}{∥}_{F}^{2}+\lambda_{u}∥\sigma u{∥}_{0}+\lambda_{v}∥\sigma v{∥}_{0}\\ \text{subject to}\;∥u{∥}_{2}=1,∥v{∥}_{2}=1. \end{array}$$

where the **v** vector is of size *d*_1_+*d*_2_. In comparison with Eq.(3), Eq.(4) uses a single *σ* for the two views, and the concatenated **v** is constrained to be a unit vector rather than individual **v**_1_ and **v**_2_. It is easy to show that any optimal solution to Problem (3) can become a feasible solution to Problem (4) by properly rescaling **v**_1_ and **v**_2_ and absorbing the scaling factors by *σ*_1_ and *σ*_2_ to make *σ*_1_=*σ*_2_, but is not necessarily an optimal solution to Problem (4). An optimal **v** to Problem (4) may have either **v**_1_ or **v**_2_ be zero, which is however not allowed in Eq.(3). When one of the **v** vectors is zero, the resultant clusters differ only on one view of the features. As an example, we concatenated 64 clinical features to 1248 SNPs in a disease subtyping analysis. Because the genetic markers outweighed the clinical features, the resultant clusters differed significantly only on the SNPs, leading to disease subtypes that could not be clinically recognized.

### A fast algorithm for two-view joint biclustering

The proposed formulation (2), although is a mixed-integer program, can be effectively solved after proper relaxations. We design an alternating optimization algorithm to solve it by splitting the variables into three working sets: one set consists of the **u** vectors; one set consists of the **v** vectors; and the last set consists of the binary variables in **z**. We optimize the variables in one working set at a time in alternative steps. 

(1) **Find the optimal u**_**1**_**, v**_**1**_**, u**_**2**_**, and v**_**2**_** with fixed z**

When **z** is fixed, Problem (2) can be decomposed into two sub-problems that optimize with respect to each individual view. Without loss of generality, we show how to optimize **u**_1_ and **v**_1_ by solving the following sub-problem with a fixed **z**.

(5) $$\begin{array}{l} \underset{\sigma_{1},u_{1},v_{1}}{\min}\;∥M_{1}-\sigma_{1}(z⊙u_{1})v_{1}^{T}{∥}_{F}^{2}+\lambda_{v_{1}}∥\sigma_{1}v_{1}{∥}_{0}\\ \text{subject to}\;∥u_{1}{∥}_{2}=1,∥v_{1}{∥}_{2}=1, \end{array}$$

which can be solved by alternating between optimizing for **u** and for **v**. 

(a) *Solve for***v**_1_*when***u**_1_*is fixed*

We solve the following equivalent problem for the optimal ${\tilde{v}}_{1}$ by relaxing the unit length constraint on **v**_1_, and then setting $\sigma_{1}=∥{\tilde{v}}_{1}{∥}_{2}$ and $v_{1}={\tilde{v}}_{1}/\sigma_{1}$.

(6) $$\begin{array}{ll} \underset{{\tilde{v}}_{1}}{\min} & ∥M_{1}-(z⊙u_{1}){\tilde{v}}_{1}^{T}{∥}_{F}^{2}+\lambda_{v_{1}}∥{\tilde{v}}_{1}{∥}_{0}. \end{array}$$

Similar to the single-view SSVD, we relax the *0-norm* to have the *ℓ*_1_ vector norm, and solve for **v** by minimizing $∥M_{1}-(z⊙u_{1}){\tilde{v}}_{1}^{T}{∥}_{F}^{2}+\lambda_{v_{1}}∥{\tilde{v}}_{1}{∥}_{1}$. Each component ${\tilde{v}}_{1(j)}$ in ${\tilde{v}}_{1}$ can be computed independently from the others by solving

$$\underset{{\tilde{v}}_{1(j)}}{\min}\;\;\;\overset{2}{\underset{1(j)}{\tilde{v}}}-2\alpha_{\left(j\right)}{\tilde{v}}_{1(j)}+2\beta |{\tilde{v}}_{1,(j)}|,$$

where $\alpha_{\left(j\right)}=u_{1}^{T}M_{1(\cdot ,j)}$, and $\beta =\lambda_{v_{1}}/2$. T by soft-thresholding [12]:

(7) $$\begin{array}{ll} {\tilde{v}}_{1(j)}= & \left\{\begin{array}{ll} \alpha_{\left(j\right)}-\beta , & \text{if}\;\alpha_{\left(j\right)}>\beta ,\\ \;0, & \text{if}\;|\alpha_{\left(j\right)}|\leq \beta ,\\ \alpha_{\left(j\right)}+\beta , & \text{if}\;\alpha_{\left(j\right)}<-\beta , \end{array}\right)\;\;\;j=1,\cdots \;,\mathrm{d.} \end{array}$$

(b) *Solve for***u**_1_*when***v**_1_*is fixed*

After **v**_1_ is obtained and fixed, we optimize Problem (5) with respect to *σ*_1_ and **u**_1_. We let ${\tilde{u}}_{1}=\sigma_{1}u_{1}$, and solve the following problem to obtain ${\tilde{u}}_{1}$. By setting $\sigma_{1}=∥{\tilde{u}}_{1}{∥}_{2}$ and $u_{1}={\tilde{u}}_{1}/\sigma_{1}$, we obtain a solution to Problem (5).

(8) $$\begin{array}{ll} \underset{{\tilde{u}}_{1}}{\min} & ∥M_{1}-(z⊙{\tilde{u}}_{1})v_{1}^{T}{∥}_{F}^{2}. \end{array}$$

Each component **u**_1(*i*)_ in an optimal **u**_1_ can be independently and analytically computed as follows:

(9) $$\begin{array}{ll} {\tilde{u}}_{1(i)}= & \left\{\begin{array}{ll} \frac{M_{1(i,\cdot )}v_{1}}{z_{\left(i\right)}}, & \text{if}\;z_{\left(i\right)}\neq 0\\ \;0, & \text{if}\;z_{\left(i\right)}=0. \end{array}\right)\;\;\;i=1,\cdots \;,\mathrm{n.} \end{array}$$

(2) **Find the optimal z with fixed u**_**1**_**, v**_**1**_**, u**_**2**_**, and v**_**2**_

When all values of **u**’s and **v**’s are fixed in Problem (2), the optimization problem becomes:

(10) $$\begin{array}{l} \underset{z\in {ℬ}_{n},\sigma_{1},\sigma_{2}}{\min}\;∥M_{1}-\sigma_{1}(z⊙u_{1})v_{1}^{T}{∥}_{F}^{2}+∥M_{2}\\ \;\;\;\;\;\;-\sigma_{2}(z⊙u_{2})v_{2}^{T}{∥}_{F}^{2}+\lambda_{z}∥z{∥}_{0}. \end{array}$$

Denote the values of *σ*_*i*_’s from the previous iteration by ${\hat{\sigma }}_{1}$ and ${\hat{\sigma }}_{2}$. We temporarily relax the binary **z** variables to be real-valued and then let $\tilde{z}={\hat{\sigma }}_{1}z$. Again, we use the *ℓ*_1_-norm of $\tilde{z}$ to approximate its 0-norm and solve the following problem for $\tilde{z}$:

(11) $$\begin{array}{l} \underset{\tilde{z}}{\min}\;∥M_{1}-(\tilde{z}⊙u_{1})v_{1}^{T}{∥}_{F}^{2}+∥M_{2}\\ \;\;\;-({\hat{\sigma }}_{2}/{\hat{\sigma }}_{1})(\tilde{z}⊙u_{2})v_{2}^{T}{∥}_{F}^{2}+\lambda_{z}∥\tilde{z}{∥}_{1} \end{array}$$

The normalization step for $\tilde{z}$ by *σ*_1_ is used to contrast the different singular values for the different views so re-scaling **z** will not cause an issue. Note that Problem (11) can be rewritten as follows:

$$\begin{array}{ll} \underset{\tilde{z}}{\min} & ∥M-\text{diag}(\tilde{z})E{∥}_{F}^{2}+\lambda_{z}∥\tilde{z}{∥}_{1} \end{array}$$

where **M**=[**M**_1_**M**_2_] is obtained by concatenating the data matrices in columns, $E=[u_{1}v_{1}^{T}\;\;({\hat{\sigma }}_{2}/{\hat{\sigma }}_{1})u_{2}v_{2}^{T}]$, and $\text{diag}(\tilde{z})$ converts $\tilde{z}$ into a diagonal matrix. Then, each component of an optimal $\tilde{z}$ can be analytically computed as follows:

(12) $$\begin{array}{ll} {\tilde{z}}_{\left(i\right)}= & \left\{\begin{array}{ll} \gamma_{\left(i\right)}-\theta , & \gamma_{\left(i\right)}>\theta \\ \;0, & |\gamma_{\left(i\right)}|\leq \theta \\ \gamma_{\left(i\right)}+\theta , & \gamma_{\left(i\right)}<-\theta \end{array}\right)\;\;\;i=1,\cdots \;,\mathrm{n.} \end{array}$$

where $\gamma_{\left(i\right)}=\frac{E_{\left(i,\cdot \right)}M_{\left(i,\cdot \right)}^{T}}{∥E_{\left(i,\cdot \right)}{∥}_{2}^{2}}$ and $\theta =\frac{\lambda_{z}}{2∥E_{\left(i,\cdot \right)}{∥}_{2}^{2}}$. Eq.(12) is derived based on the same calculation in [12] which was used to derive Eq.(7).

After obtaining $\tilde{z}$, the solution **z** to Problem (10) can be calculated as follows:

(13) $$\begin{array}{ll} z_{\left(i\right)}= & \left\{\begin{array}{ll} 1, & \text{if}\;{\tilde{z}}_{\left(i\right)}\neq 0\\ 0, & \text{if}\;{\tilde{z}}_{\left(i\right)}=0. \end{array}\right)\;\;\;i=1,\cdots \;,\mathrm{n.} \end{array}$$

To preserve the same objective value of Problem (2) after updating **z**, we update **u**_1_ and **u**_2_ as follows:

(14) $$\begin{array}{ll} u_{\left(i\right)}= & \left\{\begin{array}{ll} u_{\left(i\right)}/{\tilde{z}}_{\left(i\right)}, & \text{if}\;{\tilde{z}}_{\left(i\right)}\neq 0,\\ \;0, & \text{if}\;{\tilde{z}}_{\left(i\right)}=0, \end{array}\right)\;\;\;i=1,\cdots \;,\mathrm{n.} \end{array}$$

and *σ*_1_, *σ*_2_ are recalculated as: *σ*_1_=∥**u**_1_∥_2_, $\sigma_{2}=({\hat{\sigma }}_{2}/{\hat{\sigma }}_{1})∥u_{2}{∥}_{2}$; then we normalize **u**_1_ and **u**_2_ by **u**_1_=**u**_1_/∥**u**_1_∥_2_, and **u**_2_=**u**_2_/∥**u**_2_∥_2_.

The proposed algorithm alternates between solving the three sub-problems (6), (8) and (10) until a local minimizer is reached. The overall objective is monotonically non-increasing when minimizing each sub-problem, so the convergence of this iterative process is guaranteed. When applied to both synthetic and real world datasets, this process reached a convergent point in about 10 iterations. To derive another row subgroup, we repeat the algorithm using new matrices **M**_1_ and **M**_2_ that either exclude the rows corresponding to the subjects in the identified subgroup or are deflated by subtracting the identified singular value components *σ***uv**^*T*^. By repeating this procedure, the desired number of subject groups can be achieved.

### Extension to more than two views

In some applications, more than two views of data can be available. For example, besides data on clinical features and genetic markers, gene expression data may also be used in the analysis. The optimization problem (2) can be readily extended to incorporate *m* separate data matrices, e.g., **M**_*i*_, *i*=1,·,*m*, as follows:

$$\begin{array}{l} \underset{z,\sigma_{i},u_{i},v_{i},i=1,\ldots ,m\;}{\min}\overset{m}{\underset{i=1}{\sum }}∥M_{i}-\sigma_{i}(z⊙u_{i})v_{i}^{T}{∥}_{F}^{2}+\lambda_{z}∥z{∥}_{0}\\ \;\;\;\;\;\;\;+\overset{m}{\underset{i=1}{\sum }}\lambda_{v_{i}}∥\sigma_{i}v_{i}{∥}_{0},\\ \;\text{subject to}\;∥u_{i}{∥}_{2}=1,∥v_{i}{∥}_{2}=1,i=1,\ldots ,m,\\ \;\;z\in {ℬ}_{n}. \end{array}$$

This problem can be solved similarly by decomposing it into several sub-problems and solving each sub-problem in turn. We obtain the singular vectors of the data matrix in the view *i*, i.e., **u**_*i*_ and **v**_*i*_ while fixing **z** and other **u**’s and **v**’s by optimizing:

$$\begin{array}{ll} \underset{\sigma_{i},u_{i},v_{i}}{\min} & ∥M_{i}-\sigma_{i}(z⊙u_{i})v_{i}^{T}{∥}_{F}^{2}+\lambda_{v_{i}}∥\sigma_{i}v_{i}{∥}_{0},\\ \text{subject to} & ∥u_{i}{∥}_{2}=1,∥v_{i}{∥}_{2}=1. \end{array}$$

Note that when **z** is fixed, the optimization of **u**_*i*_ and **v**_*i*_ is independent from one another among different views. Thus, these singular vectors can be computed in parallel, which can reduce the computation time significantly when more computational resources are available. When **u**_*i*_ and **v**_*i*_ are fixed for all views, we solve the following problem to obtain $\tilde{z}$ and rescale $\tilde{z}$ to obtain **z**:

$$\begin{array}{ll} \underset{\tilde{z}}{\min} & \overset{m}{\underset{i=1}{\sum }}∥M_{i}-({\hat{\sigma }}_{i}/{\hat{\sigma }}_{1})(\tilde{z}⊙u_{i})v_{i}^{T}{∥}_{F}^{2}+\lambda_{z}∥\tilde{z}{∥}_{1}. \end{array}$$

Algorithm 1 summarizes all of the related steps to solve a multi-view SVD. Again, this algorithm can be repeated to obtain subsequent clusters in iterations. Although a good initialization can be problem-specific, we chose to initialize **z** with a vector of all ones, which assumes that all subjects have the potential to be in the cluster if no prior is given. 

## Results and discussion

We first validated the proposed method using synthetic data that were simulated with known cluster and association structures. We then evaluated our approach on a real world disease dataset aggregated from multiple genetic studies of cocaine dependence (CD).

Normalized mutual information (NMI) was used to measure the agreement between any two cluster solutions. Denote two clusterings by $C^{\left(1\right)}$ and $C^{\left(2\right)}$ where each clustering contains a number of clusters as a partition of a given sample, and $C_{i}$ is a set containing indexes of the subjects in the *i*-th cluster. NMI computes the *mutual information* between the two clusterings normalized by the cluster entropies. In other words,

(15) $$\begin{array}{c} \text{NMI}(C^{\left(1\right)},C^{\left(2\right)})=\frac{I(C^{\left(1\right)},C^{\left(2\right)})}{(H(C^{\left(1\right)})+H(C^{\left(2\right)}))/2} \end{array}$$

where $I(C^{\left(1\right)},C^{\left(2\right)})=\underset{i,j}{\sum }\frac{\left|C_{i}^{\left(1\right)}\cap C_{j}^{\left(2\right)}\right|}{n}\log\frac{n|\overset{\left(1\right)}{\underset{i}{C}}\cap C_{j}^{\left(2\right)}|}{\left|\overset{\left(1\right)}{\underset{i}{C}}||C_{j}^{\left(2\right)}\right|}$, $H(C)=-\underset{i}{\sum }\frac{\left|C_{i}\right|}{n}\log\frac{\left|C_{i}\right|}{n}$, and $\left|C_{i}\right|$ denotes the number of subjects in the cluster $C_{i}$. Because the true clusters are known in synthetic data, we computed NMI to measure the agreement between the true cluster assignments and the cluster assignments resulting from cluster analysis. A higher NMI value indicates better performance.

In addition to NMI, for each clustering, classifiers were constructed based on genetic markers to separate subjects in different clusters. We used the Area Under the receiver operating characteristic Curve (AUC) [15] in a 10-fold cross-validation setting to measure the genetic separability or homogeneity of the clusters in a clustering and compared it between different clusterings. We used a regularized logistic regression [16] as the classification model in these experiments.

We compared the proposed approach extensively against biclustering and multi-view analytics. We calculated NMI for different methods on synthetic data and AUC values on both synthetic and real world data. Our comparison study included the following existing methods: 

•**Single-view SSVD:** Clusters were included in the comparison by running the method of SSVD-based biclustering in the clinical view, as the biclustering method does not handle multiple views. Applying this method to genetic data created completely different clusters from those obtained in the clinical view.

•**Co-regularized spectral:** This method was proposed previously [9] to find consistent row clusters across multiple views by applying spectral clustering to each view in turn together with a co-regularization factor applied to the cluster indicator vector.

•**Kernel addition:** Radial basis function (RBF) kernels were calculated for each view and combined by summing them. Then spectral clustering was applied to the combined kernel to obtain row clusters.

•**Kernel product:** This is the same procedure as in the kernel addition described above except that kernel matrices were combined by multiplying their components in the same position.

•**Feature concatenation:** Data from the two views were combined by feature concatenation and a kernel matrix was computed based on the combined features. It was then used in spectral clustering to obtain row clusters.

### A simulation study

Two disease subtypes, *subtype 1* and *subtype 2*, were simulated. Each of the subtypes was both defined by a set of phenotypic/clinical features and associated with a set of genetic markers. However, the clinical features and genetic markers differed for the two subtypes. Thus, each subtype corresponded to a cluster of subjects with the specific clinical features and the associated SNP markers (here we assumed that minor alleles at each locus were risk variants). The goal of the simulation was to create a reference partitioning of subjects in both views (i.e., genetic markers and clinical features).

Genetic data were obtained from the 1000 Genome Project [17], in which 1092 subjects were genotyped for several million genetic markers. We randomly selected 1000 markers from chromosome 5 that had a minor allele frequency of at least 5% as genetic inputs in our experiments. Ten markers (different for each subtype) were randomly chosen to be associated with each subtype. Thus, a cluster of subjects was formed for each subtype, and we assigned subjects to a cluster if they had ≥8 risk variants out of the 10 SNPs chosen for that subtype. This amounts to an additive genetic model for each subtype (i.e., derived by adding the risk variants). Subjects who did not belong to either of the subtypes were treated as controls, forming the third subject cluster. We removed from the analysis subjects who belonged to both subtypes to ensure clarity in the partition. A total of 1013 subjects were retained. Of these, 247 and 167 were assigned to *subtype 1* and *subtype 2*, respectively, and 599 were controls. We named these clusters the genotypic clusters.

We then created clusters of the same subjects in the clinical view to be consistent to a certain degree with the genotypic clusters. Note that many diseases, although highly heritable, are multifactorial genetically and environmentally. To reflect the environmental effects on the clinical features, we introduced random noise to the synthesized clinical data so that the clinical clusters were not exactly the same as the genotypic clusters, so as to test the robustness of the proposed approach. We used a parameter *e* to indicate the relative effect that genetic variation contributed to the phenotypic variation. Denote $r_{i}^{j}$ the number of risk variants of *subtype j* shared by subject *i*, so $0\leq r_{i}^{j}\leq 10$ according to our definition of genotypic clusters. If $r_{i}^{j}*e+N(0,1)>7.5*e$, we assigned subject *i* to *subtype j*. This process created clusters of subjects that were different but similar to the genotypic clusters (with the parameter *e* reflecting the level of similarity).

We named these clusters the phenotypic clusters because they were used to synthesize clinical features such that the clinical data represented these clusters. Similarly, we removed from the analysis subjects that overlapped in the two phenotypic clusters. Fewer than 15 subjects were excluded in any simulated dataset in the experiments. In addition to these two phenotypic clusters, two additional phenotypic clusters, independent of any genetic variant and based on clinical features only, were created to make the simulated data more difficult but more realistic. Each of the two additional clusters included 200 subjects that were randomly selected among the controls. This design aimed to reflect the observation that multiple clinical clusters may exist in a sample, but only some clusters (two in our simulations) are associated with genetic factors.

We simulated 10 binary phenotypic/clinical features that exhibited the phenotypic clusters. A subject was assigned a value of 0 or 1 for each of the features according to a pre-defined probability. *Subtype 1* and *subtype 2* each were associated with three features. Subjects in each simulated phenotypic cluster were assigned a value of 1 with probabilities of 0.6, 0.5, and 0.4, respectively, for the three designated features. Each of the two additional phenotypic clusters was associated with two features, and subjects in each of the two subtypes were assigned a value of 1 in the two features, with probabilities of 0.6 and 0.5, respectively. A subject was assigned a value of 1 with a probability of 0.1 on any other features.

To evaluate how the proposed method performed when the genetic effect varied, four phenotypic datasets with *e*=1, 0.8, 0.6, and 0.4 were generated and analysed. The genetic effect on phenotypic variation decreases with decreasing *e*, which leads to a lower level of agreement between genotypic and phenotypic clusters.

All of the available methods were used to obtain three subject clusters. Table 1 provides the NMI calculated by comparing subject clusters obtained from each approach to the simulated phenotypic clusters. The proposed method has the highest NMI on all four of the datasets. With decreasing *e*, the NMI obtained by the proposed method decreases gradually, as expected, but the subject clusters consistent between the two views can still be discerned.

**Table 1:** Comparison of different methods on their cluster validity in the simulation

	***e=1***	***e=0.8***	***e=0.6***	***e=0.4***
Single-view SSVD	0.0821	0.1798	0.2432	0.2286
Co-regularized Spectral	0.2306	0.2477	0.2338	0.2549
Kernel addition	0.2587	0.2295	0.2350	0.2566
Kernel product	0.1917	0.2432	0.2302	0.2310
Feature concatenation	0.1569	0.1576	0.1532	0.1211
Proposed method	*0.7949*	*0.7693*	*0.6815*	*0.6329*

The normalized mutual information (NMI) values are shown, measuring the agreement between the clusters resulting from an approach and the simulated phenotypic clusters. The genetic contribution to the phenotypic variation varied according to different *e* values. A greater *e* value indicates a higher agreement between the simulated phenotypic clusters and genotypic clusters, making it easier for a clustering approach to recover the simulated phenotypic clusters. Italic fonts indicate the best performance in the experiments with each of the *e* values.

**Figure 1:**
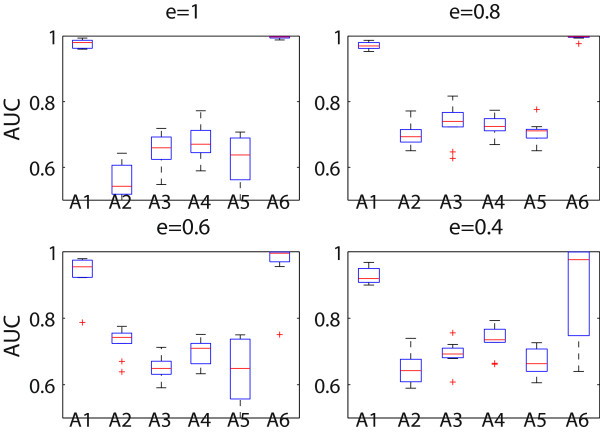
**Comparison of different methods on AUC values in the simulation.** The box plot of AUC values obtained from all approaches in the comparison is shown for the simulated data. The methods were: A1 - the proposed method, A2 - single-view SSVD, A3 - co-regularized spectral clustering, A4 - kernel addition, A5 - kernel product, and A6 - feature concatenation. The parameter *e* reflects the level of genotypic effect to the phenotypic variation in the simulated data. The AUC values characterize the genetic separability of the clusters resulting from each method.

For each cluster solution, two classification models were built to separate subjects in each of the two subtypes from controls. The subject cluster from each method containing the largest number of controls was considered the control group. The average AUC values and their interquantiles obtained by all compared approaches on each dataset are plotted in Figure 1. The proposed method achieved the second best performance on this measurement. Although the feature concatenation method obtained the clusters that were most separable genetically (i.e., with the best AUC), the clusters were not clinically recognizable. As shown in Table 1, they were the most disparate from the simulated true phenotypic clusters.

A significant advantage of the proposed method is that it can simultaneously identify the features that specify the subject clusters. We calculated the number of features that were correctly and incorrectly identified by the proposed method to measure its performance in this regard. The results are summarized in Table 2, which shows that our approach correctly identified all true associated features in both views with a very low false discovery rate (∼15/1000) when taking into account the total number of features used in the analysis.

**Table 2:** The features identified by the proposed method in both views in the simulation

		**Phenotypic view**			**Genotypic view**		
		**TF**	**TPF**	**FPF**	**TF**	**TPF**	**FPF**
*Subtype 1*	*e*=1	3	3	1	10	10	4
	*e*=0.8		3	1		10	5
	*e*=0.6		3	2		10	15
	*e*=0.4		3	0		10	10
*Subtype 2*	*e*=1	3	3	0	10	10	4
	*e*=0.8		3	0		10	4
	*e*=0.6		3	0		10	2
	*e*=0.4		3	0		10	5

The parameter *e* reflects the level of genotypic effect to the phenotypic variation in the simulated data. TF is the number of True Features used in the simulation to specify a subject cluster. TPF (True Positive Features) is the number of features correctly identified. FPF (False Positive Features) is the number of features incorrectly identified.

### A disease study: cocaine use and related behaviors

A total of 1,474 African Americans were phenotyped and genotyped for genetic studies of cocaine dependence (CD) [18]. Subjects were recruited from the Yale University School of Medicine, University of Connecticut Health Center, University of Pennsylvania School of Medicine, McLean Hospital and Medical University of South Carolina. All subjects gave written, informed consent to participate, using procedures approved by the institutional review board at each participating site. Subjects were phenotyped using a computer-assisted interview, called the Semi-Structured Assessment for Drug Dependence and Alcoholism (SSADDA) [19], a polydiagnostic instrument that was used to generate diagnoses of dependence on cocaine and other substances. Sixty-four yes-or-no variables were generated by this survey, which were also used in previous genetic association studies [1,20,21]. These variables were used as the phenotypic features. Of the 1,474 subjects, 1,287 were diagnosed with cocaine dependence. Subjects were genotyped for 1,350 SNPs selected from 130 candidate genes [4] and 186 ancestry informative markers (AIMs) using the Illumina GoldenGate Assay platform (Illumina, Inc., San Diego, CA).

The original dataset aggregated from two studies was preprocessed with a sequence of steps for data cleaning and to address population stratification. Race was classified using STRUCTURE v2.3 [22] and AIMs, which stratified the study subjects into two population groups: African Americans (AAs) and European Americans (EAs). The AA group was used in the present analysis. Of the 1,474 AAs, 93.78% had AA as their self-reported race. We excluded other population groups from the analysis. Principal components analysis (PCA) was performed on the 186 AIMs for the stratified AA population. The first PCA dimension was used in the subsequent association tests as a covariate to correct for the residual population structure. SNPs for which data were available for less than 95% of the subjects, or for which the P value for Hardy-Weinberg equilibrium was less than 10^−7^, were excluded from our analysis. The minor allele frequency (MAF) of each SNP was calculated within this AA population group. SNPs with a MAF <1*%* were removed. The remaining 1,248 SNPs were used as the genetic markers in the multi-view biclustering experiment. The SNPs selected by the proposed Algorithm 1 were then used in the association test that was based on the logistic regression model.

**Figure 2:**
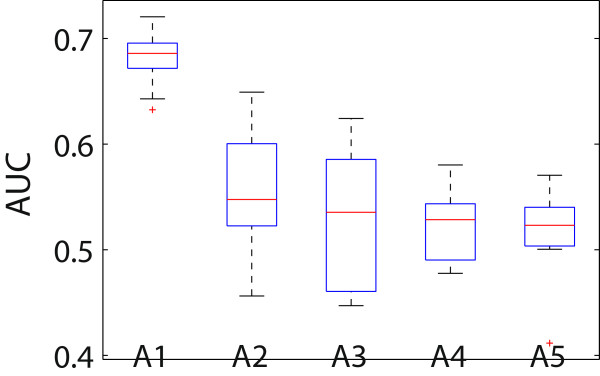
**Comparison of different methods on AUC values in the CD study.** The box plot of AUC values were obtained from all methods on the data of cocaine use and related behaviors. A1 - the proposed method, A2 - single-view SSVD, A3 - co-regularized spectral clustering, A4 - kernel addition, A5 - kernel product.

The feature concatenation method overlooked the information in the clinical or phenotypic view as observed in both the simulation study and the case study. Thus, we excluded the feature concatenation method from further comparisons. Three subject clusters were obtained from each of the methods in the comparison. Logistic regression models were built with sex, age and the first PCA dimension as covariates and tested in a manner similar to that used for synthetic data. Figure 2 shows the box plot of the AUC values. As shown there, our approach significantly outperformed all other methods with respect to the genetic separability of the resultant clusters. A paired *t*-test to compare the AUC values from our method with each of the other methods yielded a *p*-values < 0.05 for all comparisons.

**Figure 3:**
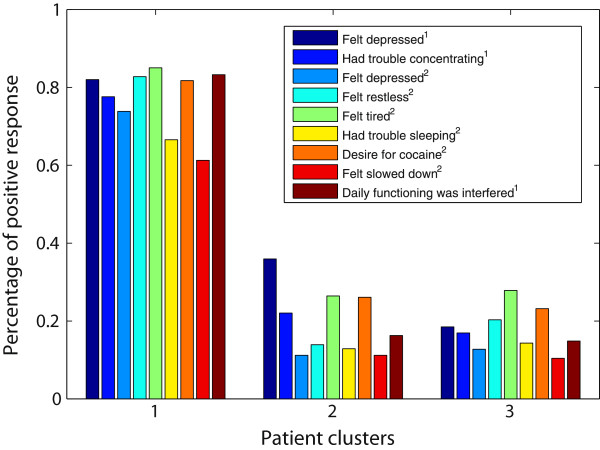
**Comparison among the three cocaine user groups on the features identified for*Group 1*.** Cocaine use symptoms are identified by the superscript ^1^, and the symptoms due to stopping, cutting down or going without cocaine are identified by the superscript ^2^. The percentage of individuals endorsing any of the features are reported for each user group.

**Figure 4:**
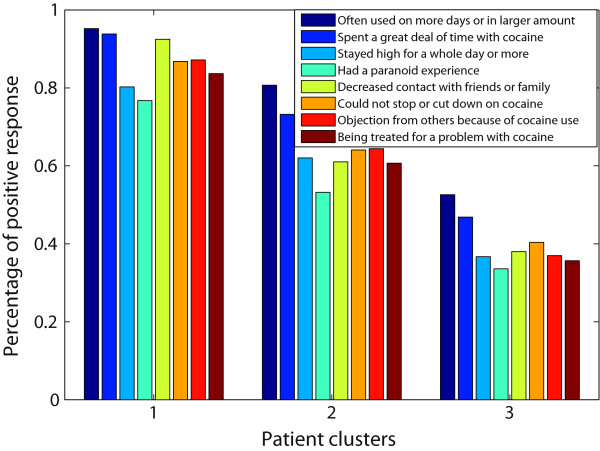
**Comparison among the three cocaine user groups on the features identified for *Group 2 *.** The percentage of individuals endorsing any of the features are reported for each user group.

For the proposed method, the three identified subject clusters contained 795 (*Group 1*), 295 (*Group 2*) and 384 (*Group 3*) subjects. *Group 1* and *Group 2* were identified consecutively, and *Group 3* contained the remaining subjects. *Group 3* contained more than 80% of the control subjects; thus, we used this group as a control group in our association analysis. The number of clinical features identified as associated with *Group 1* and *Group 2* were 18 and 17, respectively. Figures 3 and 4 compare the three subject clusters on the percentage of positive responses to the identified clinical features. A few identified features are not shown in the figures, because they are highly correlated (*r*>0.7) with the features shown.

From these two figures, we can see that *Group 1* is distinctively associated with several withdrawal symptoms, such as feeling depressed, restless, or tired when the subject stopped, cut down or went without cocaine. When *Group 2*, the second row cluster, was identified, the corresponding column cluster contained 17 clinical features. We plotted the percentage of positive responses to eight of these features for all three cocaine user groups in Figure 4. Subjects in both *Group 2* and *Group 1* showed high values on these features. Note that subjects in *Group 1* were excluded when the second cluster was derived. From these observations, we can conclude that *Group 1* is a heavy user group with many negative consequences of cocaine use, *Group 2* is a moderate cocaine user group, and *Group 3* is a low cocaine user group.

There were 114 and 237 genetic markers identified for *Group 1* and *Group 2*, respectively, by Algorithm 1. Based on these markers, two logistic regression models were built to identify the markers that had the highest predictive power in distinguishing subjects in *Group 1* or in *Group 2*, from those in the control group. Table 3 gives the 5 SNPs that received the largest magnitude of weights in the models. It is interesting to note that the *HTR2C* gene was significantly associated with *Group 1* in our study (*p*-value <10^−5^), having previously been identified with a heavy use, early-onset and high comorbidity subtype of cocaine dependence [20].

**Table 3:** Top five SNPs associated with each of the two CD subtypes

	**SNP**	**Chr**	**MAF**	**HWE**	**Gene**
	rs6318	chrX	0.3643	1.00	*HTR2C*
*Group 1*	rs2427400	chr20	0.1280	0.22	*NTSR1*
vs.	rs460401	chr21	0.3500	0.18	*GRIK1*
*Group 3*	rs10485058	chr06	0.0585	0.38	*OPRM1*
	rs2279423	chr15	0.0237	0.81	*CHRM5*
	rs897692	chr11	0.3972	0.86	*HTR3A*
*Group 2*	rs9996854	chr04	0.5436	0.61	*GABRB1*
vs.	rs481036	chr01	0.5582	0.21	*CHRM3*
*Group 3*	rs6092933	chr20	0.2070	0.17	*SLC32A1*
	rs9371781	chr06	0.3687	0.49	*OPRM1*

The five SNP markers that received the largest magnitude of weights in the two classification models that separate the subtype cases, in *Group 1* and *Group 2*, respectively, from the controls in *Group 3*. The SNP name, the SNP location (chromosome i.e., Chr), the name of the gene (Gene), the minor allele frequency (MAF) and the P-value for Hardy Weinberg equilibrium (HWE) are provided for each SNP.

## Conclusion

It is challenging to identify the genetic causes of complex disorders such as substance dependence, due to their heterogeneous clinical manifestations and complex genetic etiologies, which include gene x environment interactions. Phenotype refinement that leads to homogeneous subtypes is a promising approach to solve this problem [1,5,23-25]. However, most of the methods used to refine phenotypes take into consideration only the phenotypic information, despite the availability of genotypic information in genetic studies of a complex disorder. Thus, existing approaches have had limited success in finding a phenotypic subtype that is genetically homogeneous. In this paper, we propose a multi-view biclustering approach to refine the phenotype by jointly taking into account genetic and phenotypic information.

The proposed method is distinct from existing multi-view data analytics in that the relevant features can be identified at the same time that a subtype is determined, which is critical to its success. This increases the likelihood of finding genetic associations. The proposed method is distinct from existing biclustering methods in that it harmonizes the subject groupings in two or more views. The developed algorithm is highly scalable with large datasets because at each iteration it calculates closed-form solutions for different groups of working variables. The results from extensive experimental comparisons on both synthetic data and real world datasets demonstrate the effectiveness and superior performance of the proposed approach.

This study has a number of limitations. The proposed multi-view biclustering method, in its current form, does not simultaneously handle population stratification and phenotype-genotype association. It may spuriously identify markers that are relevant to a disease subtype due to population structure rather than being truly associated with the specific disease. Thus, population groups need to be stratified in additional steps such as those performed in our experiments. It is desirable to extend our method to address the three-way relationship among population subgroups, genotypes and phentoypes to ensure the validity of the identified phenotype-genotype associations. Further, the proposed method was used in our empirical study to identify the first two major subgroups of subjects, for which no invalid clusters caused by random noise were identified. When larger numbers of clusters are to be identified, the two methods we designed to find subsequent clusters (by either excluding subjects in the identified subgroups or deflating singular value components from the data matrix) become susceptible to the detection of invalid clusters because singular values will decrease in subsequent decomposition. Empirical studies may be needed to examine more thoroughly the signal-to-noise pattern of the proposed method.

## Competing interests

JS and JB declare that they have no competing interests. Although unrelated to this study, HRK has been a consultant or Advisory Board Member for the following pharmaceutical companies: Alkermes, Lilly, Lundbeck, Pfizer, and Roche. He is also a member of the American Society of Clinical Psychopharmacology’s Alcohol Clinical Trials Initiative, supported by AbbVie, Ethypharm, Lilly, Lundbeck, and Pfizer.

## Authors’ contributions

JB and JS designed the algorithm and all authors designed the study together. JS implemented the algorithm in Matlab and performed the experiments. HRK provided the substance dependence datasets and helped to interpret the results. JB and JS wrote the first manuscript, and HRK revised and edited it. All authors read and approved the final manuscript.
